# Serum Levels of Arachidonic Acid, Interleukin-6, and C-Reactive Protein as Potential Indicators of Pulmonary Viral Infections: Comparative Analysis of Influenza A, Respiratory Syncytial Virus Infection, and COVID-19

**DOI:** 10.3390/v16071065

**Published:** 2024-07-01

**Authors:** Simona Iftimie, Xavier Gabaldó-Barrios, Joan Penadés-Nadal, Marta Canela-Capdevila, Rubén Piñana, Andrea Jiménez-Franco, Ana F. López-Azcona, Helena Castañé, María Cárcel, Jordi Camps, Antoni Castro, Jorge Joven

**Affiliations:** 1 Department of Internal Medicine, Hospital Universitari de Sant Joan, Institut d’Investigació Sanitària Pere Virgili, Universitat Rovira i Virgili, 43204 Reus, Spain; simona.mihaela@salutsantjoan.cat (S.I.); jopenadal@gmail.com (J.P.-N.); anafelisa.lopez@salutsantjoan.cat (A.F.L.-A.); antoni.castro@urv.cat (A.C.); 2 Department of Clinical Laboratory, Hospital Universitari de Sant Joan, Institut d’Investigació Sanitària Pere Virgili, Universitat Rovira i Virgili, 43204 Reus, Spain; ruben.pinana@salutsantjoan.cat (R.P.); maria.carcel@salutsantjoan.cat (M.C.); 3 Unitat de Recerca Biomèdica, Hospital Universitari de Sant Joan, Institut d’Investigació Sanitària Pere Virgili, Universitat Rovira i Virgili, 43204 Reus, Spain; marta.canela@iispv.cat (M.C.-C.); andrea.jimenez@urv.cat (A.J.-F.); helena.castane@iispv.cat (H.C.); jorge.joven@salutsantjoan.cat (J.J.)

**Keywords:** arachidonic acid, C-reactive protein, COVID-19, infectious diseases, influenza A, interleukin-6, respiratory syncytial virus, viral infection

## Abstract

Acute respiratory tract infections, including influenza A (FluA), respiratory syncytial virus (RSV) infection, and COVID-19, can aggravate to levels requiring hospitalization, increasing morbidity and mortality. Identifying biomarkers for an accurate diagnosis and prognosis of these infections is a clinical need. We performed a cross-sectional study aimed to investigate the changes in circulating levels of arachidonic acid, interleukin 6 (IL-6), and C-reactive protein (CRP) in patients with FluA, RSV, or COVID-19, and to analyze the potential of these parameters as diagnosis or prognosis biomarkers. We analyzed serum samples from 172 FluA, 80 RSV, and 217 COVID-19 patients, and 104 healthy volunteers. Individuals with lung viral diseases showed reduced arachidonic acid concentrations compared to healthy people, with these differences being most pronounced in the order COVID-19 > RSV > FluA. Conversely, IL-6 and CRP levels were elevated across diseases, with IL-6 emerging as the most promising diagnostic biomarker, with areas under the curve (AUC) of the receiver operating characteristics plot higher than 0.85 and surpassing arachidonic acid and CRP. Moreover, IL-6 displayed notable efficacy in distinguishing between FluA patients who survived and those who did not (AUC = 0.80). These findings may provide useful tools for diagnosing and monitoring the severity of acute viral respiratory tract infections, ultimately improving patient outcomes.

## 1. Introduction

Acute respiratory tract infections often manifest with mild symptoms that tend to resolve spontaneously. Despite this, in certain instances, they may worsen to necessitate hospitalization, exerting a considerable influence on morbidity and mortality, especially among vulnerable populations such as children and older people [1]. Respiratory syncytial virus (RSV) and various strains of influenza virus, particularly influenza A (FluA), have historically been the primary causes behind these infections [2,3]. However, the advent of the COVID-19 pandemic in early 2020 precipitated a paradigm shift, with SARS-CoV-2 assuming a dominant role in this disease spectrum, coinciding with a decreased prominence of previously prevalent viral agents. Studies conducted during this period consistently underscored the overwhelming influence of SARS-CoV-2 in respiratory infections [4].

Still, in the summer of 2022, FluA and RSV resurgence was reported, causing increased hospitalizations in children and adults [5,6,7,8,9]. A confluence of factors has likely contributed to the evolving landscape of respiratory infections. Studies have indicated a diminishing risk posed by SARS-CoV-2 over time, potentially attributed to the predominance of less virulent variants, enhanced immunity derived from previous infection or vaccination, and refinements in managing COVID-19 patients [10,11]. Presently, these three infectious agents coexist, causing diseases with similar symptoms. This situation challenges clinicians treating these patients because relying solely on clinical information for accurate virus diagnosis or treatment is often insufficient. Identifying specific biomarkers enabling a more precise diagnosis and severity assessment of viral infections than conventional measures could significantly diminish inappropriate antibiotic treatment. This achievement, in turn, could optimize clinical outcomes by minimizing toxicity and adverse events, curbing healthcare expenses related to infections, and mitigating the emergence of antimicrobial-resistant strains [12]. Hence, understanding the metabolic effects and inflammatory responses of these viruses may be crucial for differential diagnosis or prognosis and finding therapeutic targets in patients with respiratory symptoms [13,14].

Our research group has paid particular interest to studying alterations in circulating lipid levels in viral infections. Lipids mediate the interaction between viral infections and the host’s metabolic and immunological responses. Viruses enter cells through protein–lipid interactions [15,16] and are released from cells via lipid vesicles [17]. Additionally, lipids serve as bioactive molecules within the immune system, and even minor variations in their chemical structures can significantly influence immune responses [18,19]. Our previous research employed semi-targeted lipidomics, identified acylcarnitines, lysophosphatidylethanolamines, arachidonic acid, and oxylipins as the most significantly altered lipid species in COVID-19 patients compared to healthy volunteers [20]. Among these metabolites, arachidonic acid can be a promising biomarker candidate due to the availability of commercial and cost-effective ELISA measurement methods. These methods do not require sophisticated technology and could be valuable additions to day-to-day clinical practice.

Alterations in lipid metabolism may contribute to the inflammatory response triggered by viral infections. This response involves a substantial increase in the production and release of inflammatory cytokines, with interleukin-6 (IL-6) being noteworthy [21]. This molecule is a versatile cytokine produced in response to tissue damage and infections and has been extensively characterized in both human and experimental studies. Elevated systemic levels of IL-6 have been associated with severe clinical outcomes in viral infections, although it is controversial whether its role is beneficial or detrimental to the host. Indeed, while some studies emphasize the role of IL-6 in mounting an effective immune response against certain viruses, others suggest that its elevation during specific viral infections could potentially facilitate virus survival or worsen clinical outcomes [22]. One of the most extensively investigated inflammation markers is C-reactive protein (CRP). The hepatic synthesis of this protein is induced by IL-6 during the acute phase of an inflammatory/infectious process and plays a role in the recognition and clearance of foreign pathogens and damaged cells [23]. A recent study has documented variances in CRP levels among patients afflicted with FluA, RSV, and COVID-19, suggesting its usefulness in evaluating the different levels of inflammatory response in these three diseases [24].

Therefore, our study was aimed to investigate the changes in circulating levels of arachidonic acid, IL-6, and CRP in patients with FluA, RSV, or COVID-19, and to analyze the potential of these parameters as diagnosis of prognosis biomarkers.

## 2. Materials and Methods

### 2.1. Study Design

A cross-sectional study was conducted on all patients admitted for acute respiratory pathology at Hospital Universitari de Sant Joan in Reus, Spain, during the specified periods. According to the hospital’s protocols, all patients underwent testing to detect the FluA, RSV, and SARS-CoV-2 viruses. The study comprised serum samples from 469 patients, encompassing 172 cases of FluA, 80 cases of RSV, and 217 cases of SARS-CoV-2 infection. Patients diagnosed with FluA and RSV were enrolled between 1 October 2022, and 28 February 2023, while those with COVID-19 were recruited from 4 October 2020, to 23 February 2022. Our facility accommodates 367 beds devoted to hospitalization and an Intensive Care Unit with 20 beds. As a general hospital, it serves a population exceeding 175,000 inhabitants, encompassing primary care facilities and elderly residences in the region. The inclusion criterion was patients presenting to our hospital with symptoms of respiratory disease. The exclusion criteria were to be younger than 18 or have missing clinical data. We recorded clinical and demographic data from electronic medical records.

The viral agent was identified by nucleic acid amplification tests (NAAT) from swab samples or with antigen tests in some COVID-19 patients. The methods employed were the Xpert^®^ Xpress Cov-2/Flu/RSV (Cepheid, Sunnyvale, CA, USA), the Procleix^®^ Panther System (Grifols Laboratories, Barcelona, Spain), or the Panbio™ COVID-19 Ag rapid test device (Abbott Laboratories, Green Oaks, IL, USA). Patients were classified according to the recommendations of the National Institute of Health [25], as follows: mildly symptomatic if they exhibited compatible symptoms without requiring hospitalization, severely symptomatic if they presented evidence of lower respiratory disease or imaging abnormalities along with specific physiological criteria, and fatally symptomatic if they necessitated advanced respiratory support measures such as high-flow oxygen therapy, mechanical ventilation, or extracorporeal membrane oxygenation. Patients who died within 30 days post-diagnosis were classified within the fatally symptomatic group for statistical analysis. The diagnosis was made on the day of admission or onset of symptoms, and blood for this study was drawn up to 2 days after diagnosis for patients with FluA or RSV and up to 7 days for patients with COVID-19.

As a control group, we employed serum samples from 104 healthy volunteers who had no clinical or biochemical evidence of infectious disease, renal insufficiency, liver disease, neoplasia, or neurological disorders. These samples were obtained before COVID-19 pandemic from a study conducted by the epidemiology department of our university, focusing on a healthy population. Participants were recruited via telephone interviews, drawing from data obtained from the censuses of several municipalities in the area. Subsequently, each participant underwent a clinical interview and basic laboratory tests. The serum was divided into aliquots and stored at −80 °C in our biological sample bank until analyses [26].

### 2.2. Laboratory Procedures

Serum arachidonic acid concentrations were assessed by ELISA (Elabscience Biotechnology Inc., Houston, TX, USA). IL-6 levels were determined by an Elecsys^®^ IL-6 immunoassay on a Cobas e801 analyzer (Roche Diagnostics, Basel, Switzerland). CRP was measured using a latex-enhanced immunoturbidimetric assay on a Cobas c702 automated analyzer (Roche Diagnostics).

### 2.3. Statistical Analyses

Quantitative data are presented as medians and 95% confidence intervals and assessed for differences with the Mann–Whitney U test (two groups) or the Kruskal–Wallis test (more than two groups). Qualitative data are shown as numbers and percentages and differences were assessed with the χ^2^ test. The influence of clinical variables on arachidonic acid, IL-6, and CRP concentrations was analyzed by multiple regression analysis, and the diagnostic accuracy was assessed by receiver operating characteristic (ROC) curves [27]. We employed SPSS 25.0 for statistical analyses and GraphPrism 9 and R 4.3.0 programs for graphics.

## 3. Results

### 3.1. Demographic and Clinical Characteristics of the Patients and the Control Group

Patients with FluA and RSV were older than those with COVID-19 and the control group. Many patients exhibited symptoms of respiratory distress; however, individuals with FluA were more likely to present with pneumonia, whereas those with RSV infection were more prone to cough and acute respiratory failure. Comorbidities were similar, although chronic lung disease was more prevalent among FluA patients, while cancer was more common in COVID-19. Patients with COVID-19 required more admissions to the Intensive Care Unit and received a higher frequency of treatment with anticoagulants. There were no significant differences in disease severity or mortality rates among the groups (Table 1).

### 3.2. Changes in the Circulating Levels of Selected Variables in Relation to the Disease

**Figure 1:**
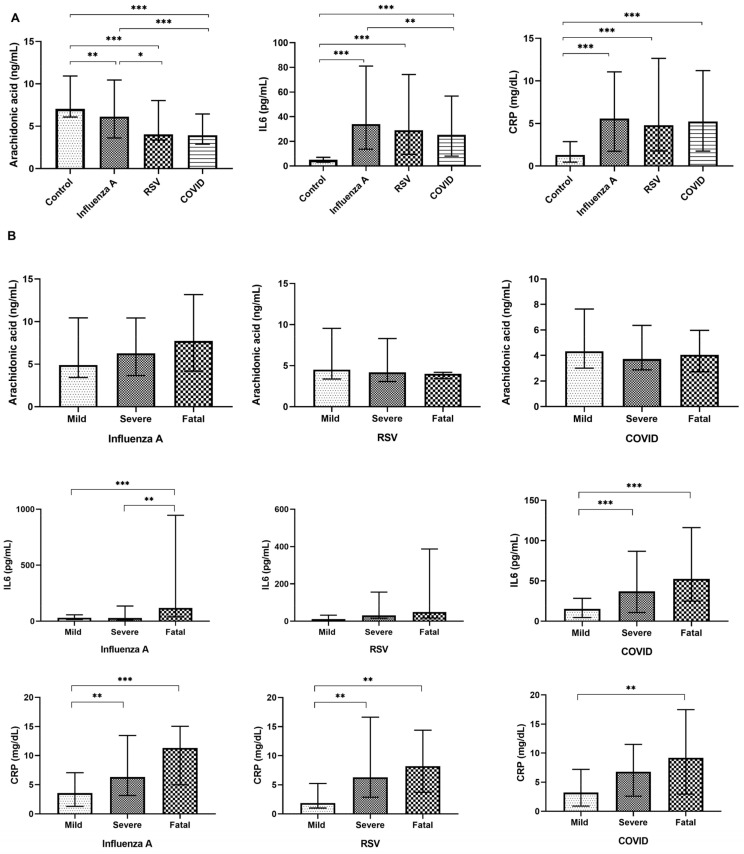
Bar plot representation illustrating the serum concentrations of arachidonic acid, interleukin-6, and C-reactive protein in healthy controls (n = 104), Influenza A patients (n = 172), respiratory syncytial virus patients (n = 80), and COVID-19 patients (n = 217), categorized by viral pathogen (**A**) and disease severity (**B**). The top and bottom lines of bars are the 25th and 75th percentiles, respectively, and the line in the middle of the bar is the median. Significance levels: * *p* < 0.05; ** *p* ≤ 0.01; *** *p* < 0.001.

The results of the analyzed variables across the studied diseases and their respective severity levels are depicted in Figure 1. Generally, individuals with lung viral diseases exhibited a notably reduced concentration of arachidonic acid compared to the control group, with these differences being most pronounced in the order COVID-19 > RSV > FluA. Conversely, they showed elevated concentrations of IL-6 and CRP, with relatively consistent levels among the diseases (Figure 1A). While serum concentrations of arachidonic acid did not show any significant differences in relation to severity in any of the studied diseases, IL-6 levels displayed a notable upward trend. Serum CRP concentrations exhibited a similar pattern, albeit less pronounced (Figure 1B).

Multiple regression analysis revealed that serum arachidonic acid concentrations were independently associated only with the presence and type of infectious disease, as indicated in Table 2. Conversely, IL-6 levels showed associations not only with the presence of disease but also with its severity and CRP concentrations (Table 3). Additionally, CRP levels showed associations with both IL-6 concentration and disease severity, albeit with slightly lower significance observed in the association with disease severity compared to IL-6, as illustrated in Table 4.

### 3.3. Serum Arachidonic Acid, IL-6, and CRP Concentrations as Disease Biomarkers

**Figure 2:**
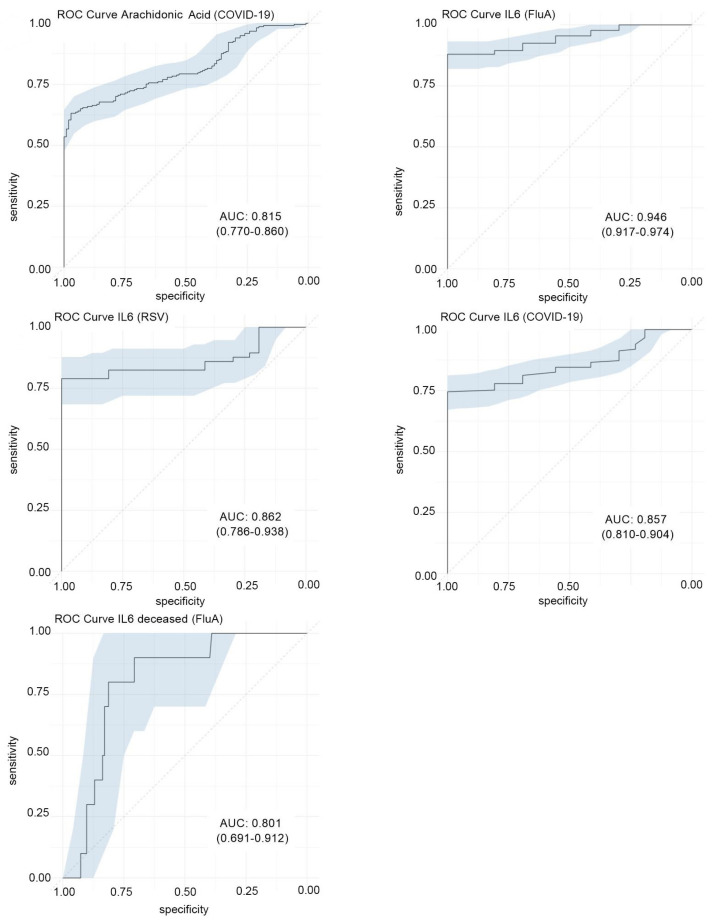
Receiver operating characteristic (ROC) curves with areas under the curve (AUC) greater than 0.80 in the distinction between patients and healthy controls and between survivors and deceased. Numbers in parentheses show the 95% CI of the AUCs. AA: arachidonic acid; FluA: Influenza A, IL6: Interleukin-6. The cutoffs of the different curves, from left to right and from top to bottom were: 6.03 ng/mL (sensitivity: 71%, specificity: 77%); 8.54 pg/mL (sensitivity = 88%, specificity = 100%); 7.35 pg/mL (sensitivity = 83%, specificity = 81%); 7.10 pg/mL (sensitivity = 78%, specificity = 81%); and 85.6 pg/mL (sensitivity = 80%, specificity = 81%).

Next, we conducted a comparative analysis of the efficacy of the studied parameters in discerning between healthy and diseased individuals (diagnosis) and forecasting disease severity (prognosis). The methodology involved employing ROC curves, with a “candidate biomarker of potential interest” designated based on an arbitrary threshold of an area under the curve (AUC) exceeding 0.80. Comprehensive AUC data are provided in Supplementary Tables S1–S3, with selected results in Figure 2.

Our findings highlight IL-6 as the most promising candidate biomarker for diagnosing viral diseases, with AUC higher than 0.85, surpassing arachidonic acid and CRP. Moreover, IL-6 displayed notable accuracy to differentiate between FluA patients who survived and those who did not (AUC = 0.80). However, none of the parameters analyzed demonstrated effective discrimination between mild cases and those categorized as severe or fatal.

## 4. Discussion

Arachidonic acid, a polyunsaturated fatty acid, is a precursor for synthesizing various inflammatory mediators, such as prostaglandins and leukotrienes. Fatty acids play indispensable roles in viral pathogenesis, serving as crucial components for membrane biosynthesis during viral replication [28]. We and other authors have previously reported a marked decrease in the circulating levels of fatty acids measured by metabolomics in patients with COVID-19 [20,29,30], and our previous study [20] showed that arachidonic acid was the fatty acid that presented the most marked alterations. The current study confirms these last results using immunoassay methods and expands them to patients with influenza A and RSV. This observation holds particular relevance from a pathophysiological standpoint, as arachidonic acid exhibits potent antiviral properties, contributing to the neutralization of enveloped viruses [31]. A decline in arachidonic acid concentrations could compromise host defense mechanisms, promoting viral persistence and replication. Indeed, exogenous supplementation with arachidonic acid effectively impedes the replication of the HcoV-229E virus in cultured cells [32]. The decrease in serum arachidonic acid levels can be explained by the need for lipids by the viral particles for their replication and an increase in their catabolism for the synthesis of inflammatory mediators. Arachidonic acid metabolites are inflammatory bioactive lipids that activate various signaling pathways, including p38 MAPK and calcium signaling pathways [33]. The observed decrease in arachidonic acid levels in the order of healthy individuals, and influenza A, RSV, and COVID-19 patients suggests a potential dysregulation of inflammatory pathways across these diseases. Therefore, the decline in arachidonic acid levels may reflect the severity of the inflammatory response mounted by the host against the respective pathogens.

In contrast, IL-6 and CRP are well-established markers of systemic inflammation and acute-phase responses, serving as sentinel indicators of host defense mechanisms activated in response to infection. These molecules actively participate in somatic maintenance processes, reflecting the organism’s investment in protecting, preserving, and repairing somatic tissues. Depending on the physiological state of the host, this maintenance effort may be directed towards resistance against pathogens, including inflammatory responses, tolerance to pathogens, harm reduction, or tissue repair mechanisms [34]. IL-6 emerges as an essential regulator of the inflammatory reaction observed in severe respiratory viral illnesses, exerting pleiotropic effects on immune cell activation, differentiation, and recruitment. The heightened levels of IL-6 in these conditions reflect the magnitude of the systemic inflammatory burden and indicate the severity of the host’s response to the invading pathogens. Consistent with prior studies, our findings corroborate the association between elevated IL-6 levels and adverse clinical outcomes, including prolonged hospital stays, complications, and mortality among COVID-19 patients [35,36,37,38]. These findings underscore the potential clinical utility of IL-6 as an early prognosis marker and advocate for its serial monitoring post-hospitalization to assess the progression of lung infections and worsening clinical features. Indeed, there are currently no described laboratory markers capable of reflecting the severity and disease course in patients with RSV infection [39].

The differential patterns observed in arachidonic acid, IL-6, and CRP levels among the infectious disease groups suggest distinct immunological signatures associated with each pathogen. While arachidonic acid appears useful in distinguishing COVID-19, IL-6 emerges as a superior marker for diagnosing this and other infectious diseases. Furthermore, our findings suggest that IL-6 could serve as a prognostic indicator, signaling the mortality risk in FluA patients. On the contrary, the discriminatory capacity of CRP is modest compared to the other molecules. These findings align with the knowledge of the complex interplay between lipid metabolism, inflammation, and regulating the immune response in infectious diseases.

This study has its limitations. The sample size and demographics of the population studied may not fully represent the broader population affected by infectious diseases, potentially limiting the generalizability of the findings. The cross-sectional design also hinders the establishment of causality or assessment of temporal relationships between biomarker levels and disease progression. Further longitudinal studies are needed to understand the dynamic changes in arachidonic acid, IL-6, and CRP levels during infection and recovery. The impact of medical interventions on biomarker levels has yet to be fully explored, and the variability in treatment protocols among patients could confound the interpretation of biomarker data. Addressing these limitations through more extensive prospective studies with standardized methodologies and comprehensive data collection protocols will be crucial for advancing our understanding of the utility of arachidonic acid, IL-6, and CRP as biomarkers in infectious diseases.

## 5. Conclusions

Our research identifies potential biomarkers for the diagnosis and prognosis of acute respiratory tract infections, including FluA, RSV infection, and COVID-19, and underscores their practical value. These findings may equip clinicians with valuable tools for diagnosing and monitoring the severity of acute viral respiratory tract infections, thereby significantly improving patient outcomes.

## Figures and Tables

**Table 1:** Demographic and clinical characteristics of the patients and the control group.

Variable	Control Group *n* = 104	Influenza A *n* = 172	RSV *n* = 80	COVID-19 *n* = 217	*p* Value
Age	50 (38–65)	73 (53–73)	75 (59–81)	62 (47–76)	<0.001
Male sex	45 (43.3)	83 (48.3)	35 (43.8)	112 (51.6)	0.449
Smoking	33 (31.7)	26 (15.1)	7 (8.8)	32 (14.7)	<0.001
Alcohol	45 (43.3)	21 (12.2)	11 (13.8)	14 (6.5)	<0.001
Initial admission department					
Emergency	N.A.	100 (58.1)	34 (42.5)	81 (37.3)	<0.001
Internal Medicine	N.A.	42 (24.4)	31 (38.8)	59 (27.2)	
Geriatry	N.A.	21 (12.2)	8 (10.0)	0 (0.0)	
Oncology	N.A.	3 (1.7)	4 (5.0)	21 (9.7)	
Intensive Care Unit	N.A.	1 (0.6)	2 (2.5)	0 (0.0)	
Surgery	N.A.	1 (0.6)	0 (0.0)	0 (0.0)	
Traumatology	N.A.	1 (0.6)	0 (0.0)	0 (0.0)	
Anesthesiology	N.A.	1 (0.6)	0 (0.0)	0 (0.0)	
Gynecology	N.A.	0 (0.0)	1 (1.2)	5 (2.3)	
Pneumology	N.A.	1 (0.6)	0 (0.0)	2 (0.9)	
Other	N.A.	1 (0.6)	0 (0.0)	49 (22.6)	
Symptoms					
Pneumonia	N.A.	66 (38.4)	20 (25.0)	32 (14.7)	<0.001
Bronchitis	N.A.	49 (28.5)	17 (21.3)	0 (0.0)	<0.001
Cough	N.A.	114 (66.3)	61 (76.3)	95 (43.8)	<0.001
Fever	N.A.	101 (58.7)	31 (38.8)	109 (50.2)	0.011
Odynophagia	N.A.	7 (4.1)	3 (3.8)	7 (3.2)	0.905
Headache	N.A.	7 (4.1)	1 (1.2)	27 (12.4)	0.001
Anorexia or hyporexia	N.A.	4 (2.3)	2 (2.5)	5 (2.3)	0.995
Myalgia	N.A.	33 (19.2)	13 (16.3)	12 (5.5)	0.001
Arthralgia	N.A.	23 (13.4)	10 (12.5)	10 (4.6)	0.006
Acute respiratory failure	N.A.	85 (49.4)	48 (60.0)	13 (6.0)	<0.001
Comorbidities					
Diabetes mellitus	N.A.	38 (22.1)	27 (33.8)	57 (26.3)	0.145
Cardiovascular disease	N.A.	100 (58.1)	49 (61.3)	111 (51.2)	0.200
Chronic liver disease	N.A.	8 (4.7)	3 (3.8)	14 (6.5)	0.579
Chronic kidney disease	N.A.	27 (15.7)	18 (22.5)	31 (14.3)	0.228
Chronic lung disease	N.A.	50 (29.1)	37 (46.3)	25 (11.5)	<0.001
Chronic neurological disease	N.A.	36 (20.9)	13 (16.3)	31 (14.3)	0.219
Cancer	N.A.	9 (5.2)	7 (8.8)	34 (15.7)	0.003
Interventions and treatments					
Intensive Care Unit admission	N.A.	7 (4.1)	6 (7.5)	30 (13.8)	0.004
Non-invasive mechanical ventilation	N.A.	8 (4.7)	3 (3.8)	9 (4.1)	0.941
Invasive mechanical ventilation	N.A.	5 (2.9)	1 (1.3)	14 (6.5)	0.078
High-flow oxygen therapy	N.A.	3 (1.7)	0 (0.0)	20 (9.2)	<0.001
Conventional oxygen therapy	N.A.	95 (55.2)	44 (55.0)	103 (47.5)	<0.001
Anticoagulants	N.A.	31 (18.0)	17 (21.3)	88 (40.6)	<0.001
Corticosteroids	N.A.	78 (45.3)	52 (65.0)	113 (52.1)	0.007
Disease severity					
Mild	N.A.	80 (46.5)	28 (35.0)	93 (42.9)	0.344
Severe	N.A.	68 (39.5)	35 (43.8)	82 (37.8)	
Fatal	N.A.	24 (13.9)	17 (21.3)	42 (19.4)	
Deceased	N.A.	17 (9.9)	11 (13.8)	19 (8.8)	0.444

N.A.: not applicable. RSV: respiratory syncytial virus. The results of qualitative variables are shown as numbers and percentages, and statistical significances were calculated by the χ^2^ test. The results of the quantitative variable (age) are shown as medians and interquartile ranges, and statistical significance was calculated by the Kruskal–Wallis test.

**Table 2:** Multiple regression analysis of the variables associated with arachidonic acid concentrations in the patients’ groups.

Variable	B	95% CI for B	β	t	*p* Value
Age	0.014	−0.041–0.069	0.039	0.502	0.616
Male sex	−1.108	−2.726–0.509	−0.078	−1.348	0.179
Smoking	0.595	−0.573–1.764	0.095	1.002	0.317
Alcohol	0.338	−0.804–1.480	0.056	0.583	0.560
Disease *	−1.038	−1.664–−0.411	−0.203	−3.260	0.001
Interleukin-6	0.000	−0.001–0.001	0.032	0.539	0.590
C-reactive protein	0.014	−0.085–0.113	0.017	0.283	0.778
Type 2 diabetes mellitus	−0.419	−2.417–1.578	−0.025	−0.413	0.680
Cardiovascular disease	−0.526	−2.577–1.524	−0.037	−0.505	0.614
Chronic liver disease	0.099	−3.453–3.651	0.003	0.055	0.956
Chronic kidney disease	−1.184	−3.567–1.199	−0.061	−0.978	0.329
Chronic lung disease	−0.330	−2.266–1.605	−0.020	−0.336	0.737
Chronic neurological disease	0.711	−1.546–2.969	0.037	0.620	0.536
Cancer	−1.068	−3.707–1.571	−0.046	−0.796	0.426
Severity **	−0.057	−1.447–1.334	−0.006	−0.080	0.936
Deceased	−0.611	−4.141–2.919	−0.025	−0.341	0.734
Constant	8.951	4.278–13.625		3.770	<0.001

* Influenza A, respiratory syncytial virus infection or COVID-19; ** mild, severe, or fatal. Model summary: r = 0.333; standard error: 6.877; sum of squares regression = 1733.696; sum of squares total = 15,591.962; *p* = 0.004.

**Table 3:** Multiple regression analysis of the variables associated with interleukin-6 concentrations in the patients’ groups.

Variable	B	95% CI for B	β	t	*p* Value
Age	−4.832	−11.232–1.568	−0.115	−1.486	0.138
Male sex	108.827	−80.500–298.154	0.065	1.131	0.259
Smoking	−2.108	−138.983–134.767	−0.003	−0.030	0.976
Alcohol	18.935	−114.661–152.531	0.026	0.279	0.780
Disease *	−98.391	−172.101–−24.681	−0.163	−2.627	0.009
Arachidonic acid	3.679	−9.758–17.116	0.031	0.539	0.590
C-reactive protein	16.573	5.151–27.995	0.169	2.856	0.005
Type 2 diabetes mellitus	−31.376	−264.992–202.240	−0.016	−0.264	0.792
Cardiovascular disease	10.427	−229.435–250.289	0.006	0.086	0.932
Chronic liver disease	−95.142	−510.319–320.035	−0.025	−0.451	0.652
Chronic kidney disease	114.124	−163.616–392.864	0.050	0.806	0.421
Chronic lung disease	−81.901	−308.071–144.268	−0.043	−0.713	0.477
Chronic neurological disease	−13.793	−277.898–250.312	−0.006	−0.103	0.918
Cancer	29.816	−279.067–338.698	0.011	0.190	0.849
Severity **	310.316	151.692–468.940	0.270	3.850	<0.001
Deceased	−358.220	−769.013–52.574	−0.123	−1.716	0.087
Constant	−270.133	−828.788–288.522		−0.952	0.342

* Influenza A, respiratory syncytial virus infection or COVID-19; ** mild, severe, or fatal. Model summary: r = 0.351; standard error: 804.139; sum of squares regression = 26,704,617.21; sum of squares total = 216,170,131.9; *p* = 0.001.

**Table 4:** Multiple regression analysis of the variables associated with C-reactive protein concentrations in the patients’ groups.

Variable	B	95% CI for B	β	t	*p* Value
Age	0.045	−0.019–0.108	0.104	1.385	0.167
Male sex	2.538	0.677–4.400	0.149	2.684	0.008
Smoking	−0.294	−1.653–1.064	−0.039	−0.426	0.670
Alcohol	−0.408	−1.734–0.918	−0.056	−0.605	0.545
Disease *	−0.249	−0.989–0.491	−0.040	−0.662	0.508
Arachidonic acid	0.019	−0.114–0.153	0.016	0.283	0.778
Interleukin-6	0.002	0.001–0.003	0.160	2.856	0.005
Type 2 diabetes mellitus	2.233	−0.072–4.539	0.111	1.907	0.058
Cardiovascular disease	−0.566	−2.946–1.815	−0.033	−0.468	0.640
Chronic liver disease	−0.479	−4.602–3.644	−0.012	−0.229	0.819
Chronic kidney disease	−0.487	−3.257–2.283	−0.021	−0.346	0.729
Chronic lung disease	−0.086	−2.333–2.162	−0.004	−0.075	0.940
Chronic neurological disease	0.744	−1.877–3.365	0.032	0.559	0.577
Cancer	−0.690	−3.756–2.376	−0.025	−0.443	0.658
Severity **	1.985	0.387–3.583	0.169	2.444	0.015
Deceased	3.203	−0.880–7.285	0.107	1.544	0.124
Constant	−1.579	−7.131–3.973		−0.560	0.576

* Influenza A, respiratory syncytial virus infection or COVID-19; ** mild, severe, or fatal. Model summary: r = 0.417; standard error: 7.984; sum of squares regression = 3929.966; sum of squares total = 22,607.704; *p* ≤ 0.001.

## Data Availability

The datasets used and/or analyzed during the current study are available upon reasonable request to the corresponding authors.

## Supplementary Materials

The following supporting information can be downloaded at: https://www.mdpi.com/article/10.3390/v16071065/s1, Table S1: Areas under the curve of the receiver operating characteristic curves for selected metabolites in distinguishing patients from the control group; Table S2: Areas under the curve of the receiver operating characteristic curves for selected metabolites in distinguishing mild from severe/fatal patients; Table S3: Areas under the curve of the receiver operating characteristic curves for selected metabolites in distinguishing survivors from deceased patients.
