# Supplementary material for: Serum Levels of Arachidonic Acid, Interleukin-6, and C-Reactive Protein as Potential Indicators of Pulmonary Viral Infections: Comparative Analysis of Influenza A, Respiratory Syncytial Virus Infection, and COVID-19
Source: Viruses. 2024 Jul 1;16(7):1065. doi: 10.3390/v16071065 (PMC11281451; doi:10.3390/v16071065)
Supplement: Supplementary file 1 [file viruses-16-01065-s001.zip › viruses-3042937-supplementary.pdf]

Supplementary Table S1. Areas under the curve of the receiver operating characteristic curves for selected metabolites in distinguishing patients from the control group.

	AUC	95% CI
Arachidonic acid		
FluA vs. CG	0.623	0.558 – 0.689
RSV vs. CG	0.739	0.658 – 0.821
COVID-19 vs. CG	0.815	0.770 – 0.860
Interleukin-6		
FluA vs. CG	0.946	0.917 – 0.974
RSV vs. CG	0.862	0.786 – 0.938
COVID-19 vs. CG	0.857	0.810 – 0.904
C-reactive protein		
FluA vs. CG	0.792	0.739 – 0.844
RSV vs. CG	0.761	0.687 – 0.835
COVID-19 vs. CG	0.767	0.713 – 0.821

AUC: Area under the curve; CG: Control group; CI: Confidence interval; COVID: Coronavirus disease; FluA: Influenza A; RSV: Respiratory syncytial virus.

Supplementary Table S2. Areas under the curve of the receiver operating characteristic curves for selected metabolites in distinguishing mild from severe/fatal patients.

	AUC	95% CI
Arachidonic acid		
FluA mild vs. severe + fatal	0.534	0.448 – 0.620
RSV mild vs. severe + fatal	0.477	0.316 – 0.639
COVID-19 mild vs. severe + fatal	0.481	0.377 – 0.586
Interleukin-6		
FluA mild vs. severe + fatal	0.571	0.471 – 0.671
RSV mild vs. severe + fatal	0.719	0.572 – 0.866
COVID-19 mild vs. severe + fatal	0.697	0.607 – 0.786
C-reactive protein		
FluA mild vs. severe + fatal	0.691	0.600 – 0.782
RSV mild vs. severe + fatal	0.745	0.604 – 0.886
COVID-19 mild vs. severe + fatal	0.684	0.591 – 0.777

AUC: Area under the curve; CI: Confidence interval; COVID: Coronavirus disease; FluA: Influenza A; RSV: Respiratory syncytial virus.

Supplementary Table S3. Areas under the curve of the receiver operating characteristic curves for selected metabolites in distinguishing survivors from deceased patients.

	AUC	95% CI
Arachidonic acid		
FluA survivors vs. deceased	0.631	0.500 – 0.762
RSV survivors vs. deceased	0.415	0.276 – 0.553
COVID-19 survivors vs. deceased	0.524	0.395 – 0.654
Interleukin-6		
FluA survivors vs. deceased	0.801	0.691 – 0.912
RSV survivors vs. deceased	0.744	0.593 – 0.895
COVID-19 survivors vs. deceased	0.735	0.609 – 0.861
C-reactive protein		
FluA survivors vs. deceased	0.697	0.571 – 0.824
RSV survivors vs. deceased	0.704	0.557 – 0.852
COVID-19 survivors vs. deceased	0.753	0.617 – 0.889

AUC: Area under the curve; CI: Confidence interval; COVID: Coronavirus disease; FluA: Influenza A; RSV: Respiratory syncytial virus.
